# Outbreak of caliciviruses in the Singapore military, 2015

**DOI:** 10.1186/s12879-017-2821-y

**Published:** 2017-11-14

**Authors:** Freddy Jun Xian Neo, Jimmy Jin Phang Loh, Peijun Ting, Wei Xin Yeo, Christine Qiu Han Gao, Vernon Jian Ming Lee, Boon Huan Tan, Ching Ging Ng

**Affiliations:** 1 DSO National Laboratories, 27, Medical Drive, #09-01, Singapore, 117510 Singapore; 2 HQ Medical Corps, Singapore Armed Forces, HQ Medical Corps, 701 Transit Road #04-01, Singapore, 778910 Singapore

**Keywords:** Sapovirus, Norovirus, Calicivirus, Outbreak, Military, Singapore, Phylogenetics, Capsid, Gastroenteritis

## Abstract

**Background:**

From 31 August to 9 September 2015, a total of 150 military personnel at a military institution in Singapore were infected with acute gastroenteritis (AGE) with an attack rate of approximately 3%. This study aimed to determine the epidemiology of the outbreak, investigate its origins, and discuss measures to prevent future occurrences.

**Methods:**

After the AGE outbreak was declared on 31 August 2015, symptom surveys, hygiene inspections, and the testing of water, food, and stool samples were initiated. We collected 86 stool samples from AGE cases and 58 samples from food-handlers during the course of the outbreak and these stool samples were tested for 8 bacterial pathogens and 2 viral pathogens (i.e., norovirus and sapovirus).

**Results:**

We detected Sapovirus (SaV), group I Norovirus (NoV GI) and group II Norovirus (NoV GII) from the stool samples of AGE cases. Further sequence analyses showed that the AGE outbreak in August was caused mainly by three rarely reported calicivirus novel genotypes: NoV GI.7, NoV GII.17 and SaV GII.3. Control measures implemented focused on the escalation of personal and environmental hygiene, which included the separation of affected and unaffected soldiers, enforcement of rigorous hand-washing and hygiene, raising awareness of food and water safety, and disinfection of communal areas with bleach.

**Conclusions:**

This study identified both NoV and SaV as the causative agents for an AGE outbreak at a Singapore military camp in August 2015. This study is also the first to report SaV as one of the main causative agents, highlighting the importance of caliciviruses as causative agents of AGE outbreaks in the Singapore military. As there are no commercially available vaccines against caliciviruses, strict personal hygiene and proper disinfection of environmental surfaces remain crucial to prevent calicivirus outbreak and transmission.

## Background

Norovirus (NoV) and sapovirus (SaV) are important viral pathogens of human gastroenteritis in the *Caliciviridae* family [1, 2]. NoV has a single-stranded positive-sense RNA genome of approximately 7.5 kb, containing 3 open reading frames (ORFs) that encode the nonstructural proteins (ORF1), a capsid protein (VP1, ORF2) and a small capsid protein (VP2, ORF3) [1, 3]. It is genetically diverse and is sorted into 6 different genogroups (GI - GVI) [1]. The commonly detected genogroups responsible for acute gastroenteritis (AGE) in human are GI and GII, although GIV has also been associated with AGE [4]. NoV is the leading cause of viral gastroenteritis in humans globally with the NoV GII.4 variant strains causing the most infections [5–7]. It is a highly infectious pathogen that can cause relatively severe disease including vomiting and diarrhea with acute onset [8]. The incubation period ranges from 24 to 48 h and symptoms usually resolve within 2 or 3 days [8]. As such, norovirus outbreaks are very likely to occur in enclosed living quarters such as military camps, hospitals, cruise ships, nursing homes and restaurants [8–10].

SaV also belongs to the *Caliciviridae* family and can cause gastroenteritis in humans and swine [2]. Similar to NoV, SaV has a single-stranded positive-sense RNA genome of approximately 7.3 to 7.5 kb, and may have two or three ORFs [1, 3]. ORF1 encodes a polyprotein that undergoes proteolytic cleavage to form the non-structural proteins and the major capsid protein VP1. ORF2 encodes the minor structural protein VP2. ORF3 encodes a small basic protein of unknown function [1, 3]. Based on complete capsid gene (VP1) sequences, SaV strains are divided into five genogroups (GI to GV), of which GI, GII, GIV, and GV are known to infect humans [2]. Traditionally, NoV and SaV differed in their epidemiologies and host ranges. While NoV infections are common in all age groups and are responsible for about 80% of all AGE outbreaks, SaV infections on the other hand, are less common and are known to cause disease primarily in children under the age of 5 years [11–13]. However it has recently been reported to affect all age groups, causing outbreaks in healthcare settings such as hospitals and long-term care facilities [14–16]. SaV has also been detected in the military setting where the communal nature of living and training environments, alongside stressors in the field, may place military personnel at higher risk of contracting and transmitting infectious diseases [17].

On 31 August 2015, an AGE outbreak occurred at a military camp in Singapore, involving a total of 150 persons. This study aimed to determine the epidemiology of the outbreak, investigate its origins, and discuss measures to prevent future occurrences.

## Methods

### Epidemiology

On 31 August 2015, a military camp in Singapore housing an approximately 5000 military personnel reported a suspected AGE outbreak. In the Singapore military, AGE cases are defined as patients with at least three episodes of watery stool and/or two episodes of vomiting within the 24 h prior to reporting sick. These cases should also have no history of recurrent diarrhea, vomiting, nor abdominal pain. A cluster of 10 or more cases meeting these criteria, linked either by space or a common food source, across 24 h constitutes an AGE outbreak.

Epidemiology teams were sent to conduct investigations and implement control measures. Symptom surveys and hygiene inspections were conducted; and water, food, and stool samples were tested. Water samples were obtained from common water sources at food halls and accommodation areas of the affected soldiers. Food samples were obtained from the food halls in the military camp and these were samples of the food consumed by the military personnel 2 days prior to the AGE outbreak. All the food and water samples were sent to an independent laboratory for culture and testing. Stool samples of cases and food handlers were collected and sent to DSO National Laboratories within 24 h after collection for PCR testing. The number of military personnel reporting sick with AGE was monitored daily by the camp clinic. Control measures were focused on the escalation of personal and environmental hygiene, which included the separation of affected and unaffected soldiers, enforcement of rigorous hand-washing and hygiene practices, and disinfection of communal areas with bleach. We describe here the epidemiological curve, symptoms experienced by the affected soldiers, and results of food, water, and stool tests.

### Laboratory analyses

#### Human stool samples and nucleic acid extraction

86 stool samples were collected from AGE cases and 58 samples from food-handlers for diagnostic testing over the course of the outbreak. DNA/RNA extraction from stool samples was performed as described previously [18]. The stool samples were first homogenized in 1 ml sterile saline using a vortex. The homogenate was then centrifuged at 400 g for 2 min to pellet stool debris. The supernatant was transferred to a new centrifuge tube and centrifuged at 10,000 g for 2 min to pellet the bacteria and viruses following which the supernatant was discarded. The pellet was then resuspended in 180 μl of ATL buffer (QIAamp DNA mini-kit, Qiagen, Hilden, Germany) and nuclei acid extracted using the tissue protocol according to manufacturer’s instructions.

### PCR diagnostic tests for stool samples

The assays used for detecting 8 bacterial pathogens (i.e., *Bacillus cereus*, *Campylobacter jejuni*, *Clostridium perfringens*, diarrheagenic *Escherichia coli*, *Listeria monocytogenes*, *Salmonella* spp., *Shigella* spp. and *Vibrio* spp.) were a mix of those developed in-house and published assays modified into multiplex PCRs and were used routinely during AGE outbreaks [10]. The real-time reverse transcription PCR (RT-PCR) to detect NoV and SaV followed the protocols as previously described [19, 20]. All these assays were validated with test panels from various external quality assessment providers.

### RT-PCR amplification of the partial capsid sequence of norovirus and sapovirus

Reverse transcription was first performed on samples that were tested positive for both NoV and SaV with oligo(dT)_20_ primer using the SuperScript® III First-Strand Synthesis System (Thermo Fisher Scientific, Waltham, USA) according to the manufacturer’s instructions. Partial capsid sequence was amplified from NoV- and SaV-positive samples according to Kojima et al. and Hansman et al. [21, 22]. The primer pairs used in this study are shown in Table 1. Primer set G1SKF and G1SKR was used for NoV GI-positive samples while primer set G2SKF and G2SKR was used for NoV GII-positive samples. The expected size of the amplicons generated by both sets of primers is about 330 bp. SaV-specific primers used for the amplification of the partial capsid sequence were SV-F13 and SV-R13 for the first-round PCR and SV-F22 and SV-R2 for the second-round PCR. The expected size of the amplicon generated by this nested PCR is about 430 bp. The PCR products were electrophoresed on 1% agarose in 0.5× Tris-borate-EDTA (TBE) buffer and visualized by Midori Green (Nippon Genetics Europe GmbH, Düren, Germany) staining. Those amplicons of the correct size were sent for Sanger sequencing.

**Table 1:** The list of primers used for genotyping NoV and SaV

		Type	Primer	Sequence (5′ to 3′)	nt position
1	Norovirus GI	Single PCR	G1SKF	CTGCCCGAATTYGTAAATGA	5342^a^
			G1SKR	CCAACCCARCCATTRTACA	5671 ^a^
2	Norovirus GII	Single PCR	G2SKF	CNTGGGAGGGCGATCGCAA	5058 ^b^
			G2SKR	CCRCCNGCATRHCCRTTRTACAT	5401 ^b^
3	Sapovirus	1st round PCR	SV-F13	GAYYWGGCYCTCGCYACCTAC	5074 ^c^
			SV-R13	GGTGANAYNCCATTKTCCAT	5876 ^c^
		2nd round PCR	SV-F22	SMWAWTAGTGTTTGARATG	5154 ^c^
			SV-R2	GWGGGRTCAACMCCWGGTGG	5591 ^c^

^a^Nucleotide position of Norwalk virus (GenBank M87661)

^b^Nucleotide position of Lordsdale virus (GenBank X86557)

^c^Nucleotide position of Manchester virus (GenBank X86560)

H = A or C or T; K = G or T; M = A or C; N = A or C or G or T; R = A or G; S = C or G; W = A or T; Y = C or T

### Phylogenetic analysis

Nucleotide sequences amplified from NoV and SaV detected during the outbreak, together with sequences from reference strains from the NCBI database were aligned with Clustal W method using the MegAlign module of the DNASTAR software package, version 12.2.0 (Lasergene, Madison, USA). A phylogenetic tree from bootstrap analysis with 1000 replicates was generated by the Neighbor–Joining method using the MegAlign module of the DNASTAR software package, version 12.2.0 (Lasergene, Madison, USA). The phylogenetic trees were rooted using a NoV GIII strain (Aba-Z5/GIII.1/HUN/EU360814) and a Porcine SaV GIII strain (Porcine Complete Genome/NC_000940.1) for their respective trees.

## Results

### Descriptive epidemiology

**Fig. 1:**
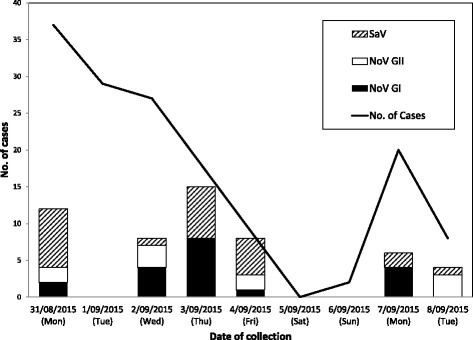
The epidemiological curve of the outbreak. The distribution of the NoV and SaV positive cases among military personnel affected by the GID outbreak between 31st August and 8th September 2015

From 31 August to 9 September 2015, an AGE outbreak affecting 150 military personnel occurred at a military camp in Singapore (attack rate of approximately 3%). These military personnel typically reside in military camp during the working week and return home on weekends if there are no military activities. Primary healthcare for military personnel is provided by clinics within the military camps, which also serve as on-site surveillance for infectious diseases. The epidemiological curve of this AGE outbreak is shown in Fig. 1. After the surge of AGE cases (*n* = 37) on the initial day of outbreak (31 August 2015), the number of new cases for the next two days remained high at *n* = 29 and *n* = 27 respectively. This is likely due to the spread of the viral pathogens before the implemented control measures can take effect. On day 4 (3 September 2015) of the outbreak, the number of cases decreased to 18, indicating that the implemented control measures are beginning to take effect. On day 5 (4 September 2015), the number cases decreased to 9 and most military personnel returned home for the weekend of 5 and 6 September 2015. Upon their return to the military camp on the 7 September 2015 (Day 8), a higher than average number of 20 AGE cases was further reported. The following day on 8 September 2015 (Day 9), the number of new cases reported was back to baseline at 8 new cases, indicating that the outbreak had been under control. After a period of careful monitoring, the outbreak was declared closed on 9 September (Day 10) with a final tally of 150 affected military personnel.

### Symptom survey of affected military personnel

A symptom survey was conducted among the patients during the outbreak and the most common clinical manifestation was diarrhea affecting 119 patients (79.3%). Other symptoms included vomiting (62.8%), abdominal pain (42.7%), nausea (42.0%) and fever (6.0%).

### Water and food surveys

Food and water samples from the involved food halls were sent for microbiological testing by an independent laboratory. Final results (data not shown) from these tests showed that all the food and water samples met satisfactory standard, suggesting that the food and water supply to the military camp are not the source of the caliciviruses that caused this AGE outbreak.

### Laboratory results

86 stool specimens collected from 65 AGE cases and 58 stool samples from the food-handlers were sent to DSO National Laboratories for molecular testing. None of the samples from the AGE cases were positive for the 8 bacterial pathogens tested. We detected a total of 21 SaV-positive samples (from 20 patients), 16 NoV GI-positive samples (from 16 patients) and 9 NoV GII-positive specimens (from 8 patients). Specimens obtained from 5 AGE patients were positive for both NoV GI and SaV. The prevalence of patients with double-pathogen infections, single-pathogen infections and non-SaV/NoV infections within these 65 AGE patients was 7.7%, 52.3%, and 40.0%, respectively. None of the stool samples from the food-handlers were tested positive for NoV or SaV, indicating that they were not the source of the caliciviruses that started the AGE outbreak. The epidemiological curve of the outbreak and the distribution of the NoV- and SaV-positive cases are shown in Fig. 1.

**Fig. 2:**
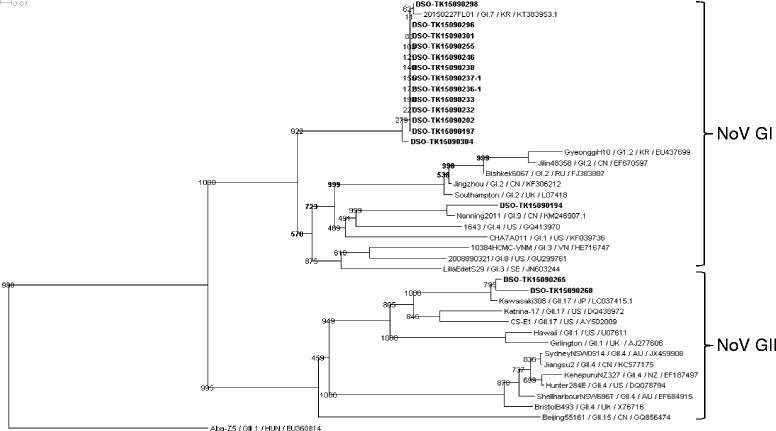
Phylogenetic analysis of norovirus (NoV) isolates based on the partial capsid nucleotide sequences (~330 bp). Phylogenetic relationships were inferred using the neighbor-joining method by MegAlign program. Percentage bootstrap values (1000 trials) of the major nodes are shown. The unit at the top of the tree indicates the nucleotide substitutions per site. The NoV isolates detected in this outbreak were highlighted in bold and named as “DSO-” followed by the sample ID. All other sequences were obtained from the NCBI public database. The NoV sequences were submitted to GenBank under accession numbers KU298647-KU2986762

Of the 25 NoV-positive samples, we were able to amplify and sequence the partial capsid region sequence from 14 NoV GI-positive samples and 2 NoV GII-positive sample. Sequences from the outbreak and reference strains from the NCBI database were aligned and a phylogenetic tree was constructed (Fig. 2). The phylogenetic tree showed that all but one of the NoV GI sequences were essentially identical to each other and clustered together with a NoV GI.7 isolate detected in stream water in Korea (20150227FL01/GI.7/KR/KT383953.1) (Fig. 2). The remaining NoV GI sequence clustered with a GI.9 isolate detected in sewage in China (Nanning2011/GI.9/CN/KM246907.1), thereby suggesting the environmental origins of the NoV GI isolates detected in this AGE episode (Fig. 2). From the frequency of the NoV GI.7 strain detected in the NoV GI-positive samples (13 out of 14 samples sequenced), it is also suggested that NoV GI.7 is the main NoV GI strain that caused the outbreak.

For NoV GII, both sequences clustered together with a human isolate of NoV GII.17 detected in Japan (Kawasaki308/GII.17/JP/LC037415.1) (Fig. 2). The remaining 7 NoV GII-positive samples failed to amplify for their partial capsid region, and no other strains of NoV GII were identified for this AGE outbreak.

**Fig. 3:**
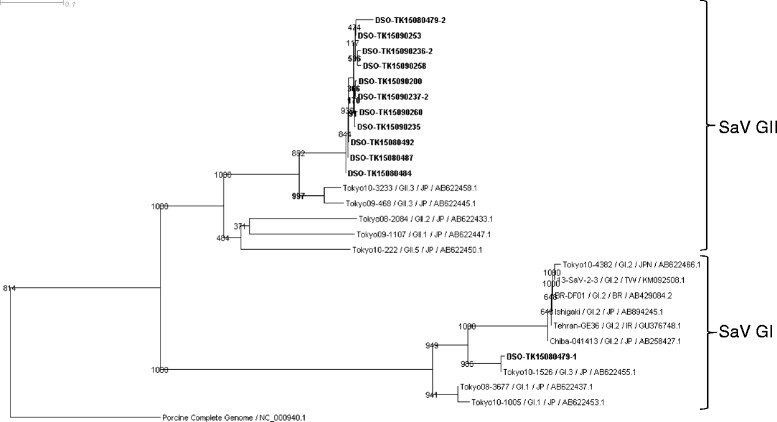
Phylogenetic analysis of sapovirus (SaV) isolates based on partial capsid nucleotide sequences (~430 bp). Phylogenetic relationships were inferred using the neighbor-joining method by MegAlign program. Percentage bootstrap values (1000 trials) of the major nodes are shown. The unit at the top of the tree indicates the nucleotide substitutions per site. The SaV isolates detected in this outbreak were highlighted in bold and named as “DSO-” followed by the sample ID. All other sequences were obtained from the NCBI public database. The SaV sequences were submitted to GenBank under accession numbers KU298663-KU298674

Of the 21 SaV-positive samples, we were able to amplify and sequence the partial capsid region sequence from 11 SaV-positive samples. Sequences from the outbreak and reference strains were aligned and a phylogenetic tree was constructed (Fig. 3). All the SaV sequences amplified were essentially identical to each other and clustered together with two SaV GII.3 human isolates detected in Japan (i.e., Tokyo09-468/GII.3/JP/AB622445.1 and Tokyo10-3233/GII.3/JP/AB622458.1), suggesting the human origin of this SaV strain. Interestingly, one of the SaV GII.3-infected patients was also simultaneously infected with another strain of SaV closely related to a human SaV GI.3 isolate detected in Japan (Tokyo10-1526/GI.3/JP/AB622455.1). From the frequency of the SaV GII.3 strain detected in the SaV-positive samples (11 out of 11 samples sequenced), it is also suggested that SaV GII.3 is the main SaV GII strain that caused the outbreak.

The NoV and SaV sequences amplified for this study were submitted to GenBank under accession numbers KU298647-KU298674.

## Discussion

This study identified both NoV and SaV as the causative agents for an AGE outbreak at a Singapore military camp in August 2015. Through rapid identification of the pathogens involved and prompt enforcement of public health measures to prevent person-to-person spread of the caliciviruses, this outbreak was quickly controlled within 10 days, resulting in an attack rate of approximately 3% which is much lower compared to those of previously reported norovirus AGE outbreaks in military settings which ranged from 8.3% to 15.0% [10, 23].

Further sequence analyses showed that this outbreak is caused mainly by three rarely-reported calicivirus genotypes: SaV GII.3, NoV GI.7 and NoV GII.17. This study is the first, to our knowledge, to report SaV as one of the main causative agents for an AGE outbreak in the Singapore military. The SaV strain associated with this outbreak was genotyped to be SaV GII.3 and clustered with two human isolates detected in Japan. Although SaV GII.3 has not been associated with any AGE outbreaks in the military, it has been detected in non-hospitalized adults with sporadic cases of AGE in China [16].

A study performed by Ueki et al. has observed that there were high sequence identities between the NoVs detected in the AGE patients and the NoVs detected in the water environment (i.e., domestic sewage, river water etc.) in the same area [24]. For the current study, all the detected NoV GI isolates clustered with environmental isolates detected in the stream water in Korea and in the sewage water in China (Fig. 2). As such, this may suggest that the NoV GI strains detected in this AGE outbreak in Singapore might have their origins from the NoV GI strains that were circulating in Korea and China. NoV GIs are not as well-studied as their GII counterparts due to their lower prevalence [7]. A recent study from Canada, however, showed an increase in the prevalence of NoV GI, with GI.6 and GI.7 as predominating strains [25]. The United States also reported increased NoV GI prevalence and ranked NoV GI.7 fourth within all AGE outbreaks occurring between 2009 to 2013 [26]. NoV GI has also been reported to cause outbreaks in Singapore military camps and other military institutions overseas [10, 27], highlighting the outbreak potential of NoV GI.

Our sequencing efforts also confirmed that two of the NoV GII positive samples belonged to genotype GII.17 and they are closely related to an emerging NoV GII.17 variant that was prevalent in Japan from December 2014 onwards [28]. Modeling of the VP1 protein of this variant has shown that it has the potential to cause large-scale AGE outbreaks, presumably by escaping host immunity and improving VP1’s capacity to bind to the histo-blood group antigens [28]. The results of the current study mirrored the global emergence of NoV GII.17 [29, 30].

Based on the investigation findings, including case distribution, food, water and stool sample testing, it was postulated that the cause of this AGE outbreak may be due to the simultaneous introduction of new emerging strains of caliciviruses into a vulnerable population living in a communal military setting. The deviation of the NoV strains (i.e., NoV GI.7 and GII.17) from the traditional outbreak-associated GII.4 strain and the introduction of the emerging SaV strain (SaV GII.3) to the military camp may have resulted in the majority of the military personnel being immunologically susceptible to falling ill with AGE. This in turn contributed to the high number of cases during the first few days of the AGE outbreak.

Currently, there is no commercially available vaccine for both NoV and SaV. Although there is a potential NoV vaccine being tested in human clinical trial [31], efforts of the NoV vaccine development had been focused mainly on the prevention of the traditionally known NoV pandemic strain GII.4 [31, 32]. The detection of emerging strains of NoV and SaV serves as reminders of the outbreak potential of these genotypes and efforts should be made to evaluate the cross-reactivity of potential norovirus vaccine candidates against these emerging strains of NoV. Efforts should also be invested to develop vaccine against SaV, which in recent years has become an important emerging pathogen [14–16].

As there is currently no routine AGE bio-surveillance program in the Singapore military, the prevalence of these strains of emerging caliciviruses among the military personnel with sporadic cases of AGE in the military remains unknown. Future studies will include widening bio-surveillance efforts to cover routine peacetime AGE surveillance so as to establish the baseline prevalence of gastrointestinal pathogens and to better understand the clinical and epidemiological patterns of these newly emerging caliciviruses.

## Conclusions

This study highlighted the importance of caliciviruses such as NoV and SaV as causative agents of AGE outbreaks in the Singapore military. As there are currently no commercially available vaccines against caliciviruses, strict personal hygiene and proper disinfection of environmental surfaces remain crucial to prevent calicivirus outbreaks and further transmission.

## Abbreviations

AGE: Acute gastroenteritis
AR: Attack rate
NoV: Norovirus
ORF: Open reading frame
SAF: Singapore Armed Forces
SaV: Sapovirus
TBE: Tris-borate-EDTA
